# The Protective Effect of Male Circumcision as a Faith Lift for the Troubled Paradigm of HIV Epidemiology in Sub-Saharan Africa

**DOI:** 10.1371/journal.pmed.0030064

**Published:** 2006-01-31

**Authors:** John J Potterat, Devon D Brewer, Stephen Q Muth, Stuart Brody

**Affiliations:** **1**Colorado Springs, ColoradoUnited States of America; **2**Interdisciplinary Scientific Research and University of WashingtonSeattle, WashingtonUnited States of America; **3**Quintus-ential SolutionsColorado Springs, ColoradoUnited States of America; **4**University of PaisleyPaisleyUnited Kingdom

Auvert and colleagues present preliminary evidence for the protective effect of male circumcision on HIV acquisition [ 1]. Their report also reveals several problems with the widely held assumption that penile–vaginal sex accounts for the overwhelming majority of HIV transmission in sub-Saharan Africa.

We are baffled that the factor most strongly associated with incident HIV infection—attendance at “a clinic for a health problem related to the genitals” (rate ratio, 5.7)—is neither highlighted nor specifically discussed. Given evidence for increased risk of acquiring HIV from treatment for sexually transmitted diseases (STDs) in sub-Saharan Africa (relative to untreated STDs) [ 2], such a context for HIV acquisition should have been more assiduously explored, especially regarding nosocomial transmission.

Regrettably, the authors did not control for blood exposures (e.g., other types of medical or dental care, including care from “street doctors” and village injectionists, injections with syringes kept at home, ritualistic procedures, and injection drug use). Nor did they assess anal intercourse, the variable most strongly associated with sexual transmission of HIV. Anal intercourse is not uncommon in sub-Saharan Africa [ 3]. The authors also did not ask participants to specify the sex of their nonspousal partners, despite much evidence for bisexual behavior on the part of many “heterosexual” men in sub-Saharan Africa [ 3].

Furthermore, the authors did not report the relationship between level of condom use and HIV incidence. The need for more detailed investigation of sexual exposures is underlined by the negligible associations between such traditional measures of sexual risk—any type of unprotected sex, the number of sexual exposures (“contacts”), and the number of nonspousal partners—and HIV incidence [ 1]. Indeed, these results replicate the frequent lack of association between sexual behavior variables and HIV incidence or epidemic trajectories in sub-Saharan Africa [ 4]. (The authors should also report HIV incidence in persons reporting no sexual activity during specified study intervals.) Of concern as well is the high per coital act–HIV transmission probability implied by the data presented. A high transmission probability would suggest that the HIV prevalence in their participants should be greater than the 4%–5% observed at baseline.

Until all modes of HIV transmission—by sex and by puncturing—are comprehensively investigated [ 5, 6], the most effective means of preventing HIV transmission will remain shrouded. In light of the anomalies and lacunae in Auvert and colleagues' study, the protective effect of male circumcision they observed amounts to a faith lift for the empirically beleaguered paradigm of heterosexual HIV transmission in sub-Saharan Africa [ 7].
